# Recurrent Post-traumatic Frontal Mucocele Following Previous Endoscopic Management: Alternative Surgical Reconstruction With Partial Capsule Resection

**DOI:** 10.7759/cureus.115069

**Published:** 2026-08-24

**Authors:** Ludmila Rodrigues Ribeiro da Silva

**Affiliations:** 1 Burn Reconstructive Surgery, Shriners Hospitals for Children, Galveston, USA

**Keywords:** capsule resection, case report, frontal sinus mucocele, frontal sinus surgery, frontal sinus trauma, mucocele excision, post-traumatic mucocele, recurrent mucocele

## Abstract

Frontal sinus mucoceles are benign but locally aggressive expansile lesions that may occur years after trauma or previous sinus surgery. Their progressive enlargement can lead to bone remodeling, facial deformity, orbital involvement, and intracranial complications. Endoscopic marsupialization is currently considered the standard treatment in most cases; however, recurrence remains possible, particularly in patients with altered anatomy secondary to trauma or previous surgical manipulation. This report describes the case of a 55-year-old man with a history of severe frontal trauma after a motor vehicle accident resulting in loss of the anterior table of the frontal sinus. The patient subsequently developed a frontal mucocele that had previously been treated elsewhere with endoscopic frontal sinus drainage and osteoma shaving. Despite initial management, recurrence occurred slightly more than one year later, with progressive frontal deformity and patient dissatisfaction. An alternative surgical strategy was therefore proposed consisting of complete frontal sinus drainage, partial capsule resection, excision of redundant frontal skin after decompression, and reduction with closure of the residual capsule. Histopathological analysis of the capsule and drained material demonstrated no evidence of neoplasia. The patient evolved with satisfactory aesthetic contour, good scar healing, and no recurrence after one year of follow-up. This case highlights an alternative surgical option for selected patients with recurrent post-traumatic frontal mucoceles and anatomically distorted frontal sinuses following previous endoscopic management.

## Introduction

Frontal sinus mucoceles are benign, epithelium-lined, expansile lesions that develop following obstruction of the frontal sinus drainage pathway. Progressive accumulation of mucus results in gradual sinus expansion and pressure-induced bone remodeling, which may lead to orbital displacement, cosmetic deformity, and, in advanced cases, intracranial extension [1-5]. Although mucoceles may develop secondary to chronic inflammation, previous sinus surgery, trauma, osteomas, or other causes of frontal recess obstruction, the frontal sinus is the most frequently affected site because of the complexity of its drainage anatomy [1-5].

A thorough understanding of frontal recess anatomy is essential for appropriate surgical planning and long-term disease control. The intricate three-dimensional anatomy of the frontal drainage pathway contributes to the technical difficulty of surgical management and increases the risk of persistent obstruction when drainage is not adequately restored [6-8]. Frontal sinus osteomas represent another important cause of secondary frontal mucoceles because they may obstruct the frontal recess and further complicate surgical treatment [9].

Post-traumatic frontal mucoceles represent a particularly challenging clinical entity because fractures, postoperative scarring, and distortion of the frontal recess may permanently alter normal sinus drainage. These lesions may remain asymptomatic for years or even decades after the initial injury before presenting with progressive frontal swelling, orbital symptoms, or cosmetic deformity [4-6].

Endoscopic marsupialization has become the preferred treatment for most frontal mucoceles because it preserves normal anatomy, avoids external scars, and is associated with high success rates and low morbidity [2,10,11]. Nevertheless, recurrence remains possible, particularly in patients with complex frontal sinus anatomy, previous surgery, severe trauma, extensive scarring, persistent frontal recess obstruction, or associated osteomas [5,6,9-13]. In carefully selected patients, osteoplastic frontal sinus surgery or alternative reconstructive approaches may still be required when endoscopic treatment is technically difficult or when recurrent disease occurs following previous endoscopic treatment [14-16].

The mechanisms responsible for recurrence after endoscopic treatment remain incompletely understood but have been attributed to persistent obstruction of the frontal sinus outflow tract, residual pathological mucosa, postoperative fibrosis, and failure to adequately address complex post-traumatic anatomical distortion [2,6,10,13-17]. Consequently, recurrent frontal mucoceles require individualized surgical planning that considers both restoration of sinus drainage and correction of the underlying anatomical abnormality.

This report describes the successful surgical management of a recurrent post-traumatic frontal mucocele following previous endoscopic treatment using an alternative reconstructive strategy consisting of complete drainage, partial capsule resection, reduction of the residual cavity, excision of redundant soft tissue, and primary closure. The case highlights a feasible surgical option for selected patients with recurrent frontal mucoceles associated with severe post-traumatic anatomical distortion.

## Case presentation

A 55-year-old man presented with progressive frontal swelling and visible forehead deformity. Several years earlier, he had sustained severe frontal trauma during a motor vehicle accident, resulting in loss of the anterior table of the frontal sinus. Following the trauma, the patient developed a frontal mucocele that was previously managed at another institution with endoscopic frontal sinus drainage and endoscopic shaving of a frontal osteoma. Despite the initial treatment, recurrence occurred slightly more than one year later, with progressive enlargement of the frontal region and increasing aesthetic concern.

Preoperative computed tomography of the face and paranasal sinuses demonstrated an expansile frontal lesion measuring approximately 6.5 × 5.0 × 4.0 cm, with soft-tissue/fluid attenuation and thick internal content without post-contrast enhancement. The lesion was associated with regional osseous remodeling and extended inferiorly toward the left frontal sinus. Areas of bony dehiscence involving the ethmoidal roof were also identified in the right frontal region. Both frontal sinuses were opacified, with an expansile effect and associated osseous remodeling, raising the radiological differential diagnosis of a frontal mucocele. Additional findings included mucosal thickening of the ethmoid, maxillary, and sphenoid sinuses, narrowing of the ethmoidal infundibula by mucosal disease, and a hyperdense lesion adjacent to the posterior right ethmoid labyrinth suggestive of an osteoma. In the context of the patient’s previous severe frontal trauma, prior surgery, recurrent frontal swelling, and imaging findings, a recurrent post-traumatic frontal mucocele was considered the leading diagnosis.

The original CT images were not available for retrospective inclusion in this report because the examination was performed at an outside institution; therefore, the radiological findings presented here were obtained from the official imaging report.

**Figure 1:**
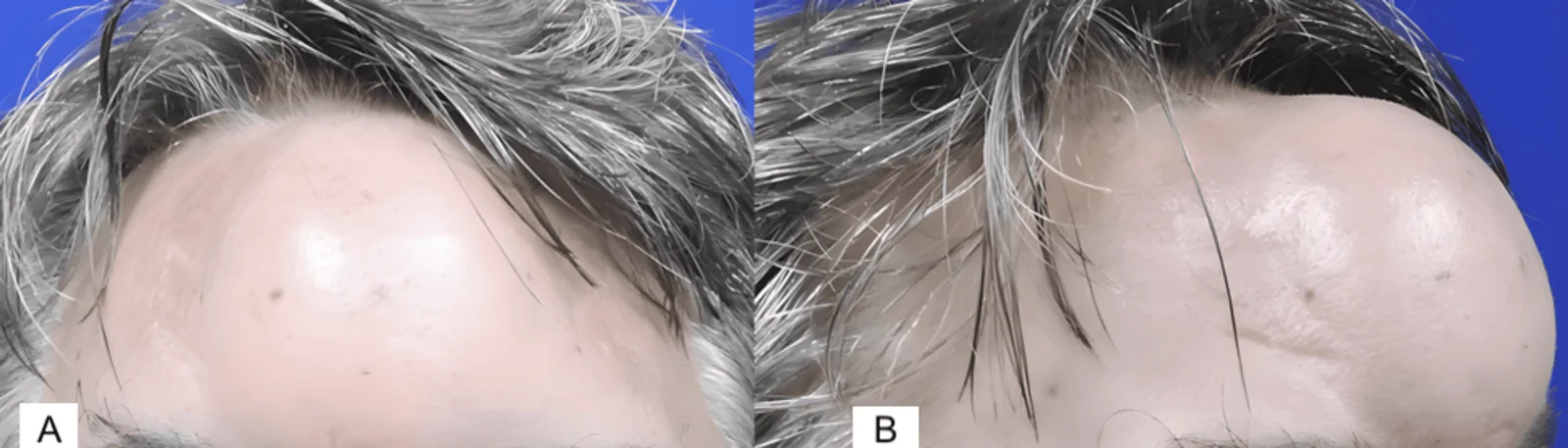
Preoperative clinical appearance of recurrent frontal mucocele following previous endoscopic treatment (A) Frontal view demonstrating prominent forehead swelling and contour deformity caused by recurrent frontal mucocele. The patient had a history of severe frontal trauma with loss of the anterior table of the frontal sinus following a motor vehicle accident and previously underwent endoscopic frontal sinus drainage with osteoma shaving at another institution. (B) Right lateral preoperative view highlighting the prominence and extent of the frontal deformity.

Physical examination demonstrated marked frontal protrusion and contour deformity involving the central forehead region (Figure 1A). Lateral evaluation demonstrated marked anterior projection and soft tissue expansion of the frontal region secondary to recurrent mucocele (Figure 1B).

**Figure 2:**
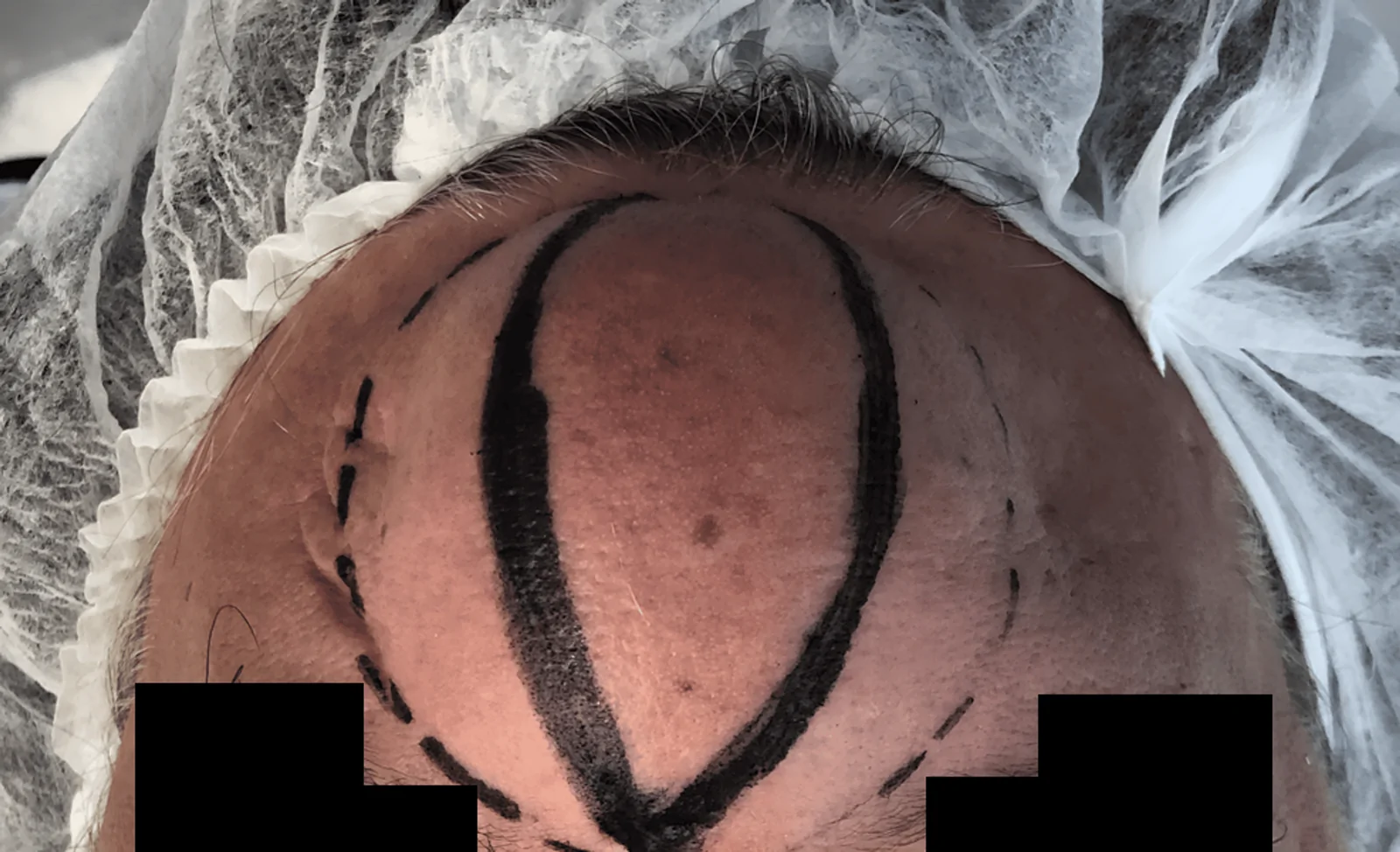
Preoperative marking for excision and reconstruction of recurrent frontal mucocele Preoperative frontal view demonstrating surgical markings for treatment of recurrent frontal mucocele in a 55-year-old male patient with a history of severe frontal trauma and prior endoscopic treatment. The markings outline the area of skin redundancy and the planned resection following decompression of the frontal lesion.

Given the large recurrent frontal lesion, previous endoscopic treatment, post-traumatic anatomical distortion, and associated osseous changes, surgical management was planned with the objective of achieving complete drainage while simultaneously addressing the persistent frontal deformity and residual cavity. Preoperative markings were designed to provide direct access to the lesion while allowing excision of redundant skin and soft tissue following decompression (Figure 2).

The diagnosis and surgical strategy were established by integrating the history of severe frontal trauma, previous endoscopic treatment, recurrent progressive frontal swelling, physical examination, and the available radiological findings. Given the large recurrent lesion, post-traumatic anatomical distortion, associated osseous changes, and recurrence following previous endoscopic treatment, direct surgical access was selected to permit complete drainage while simultaneously addressing the residual cavity and associated frontal soft-tissue deformity.

**Figure 3:**
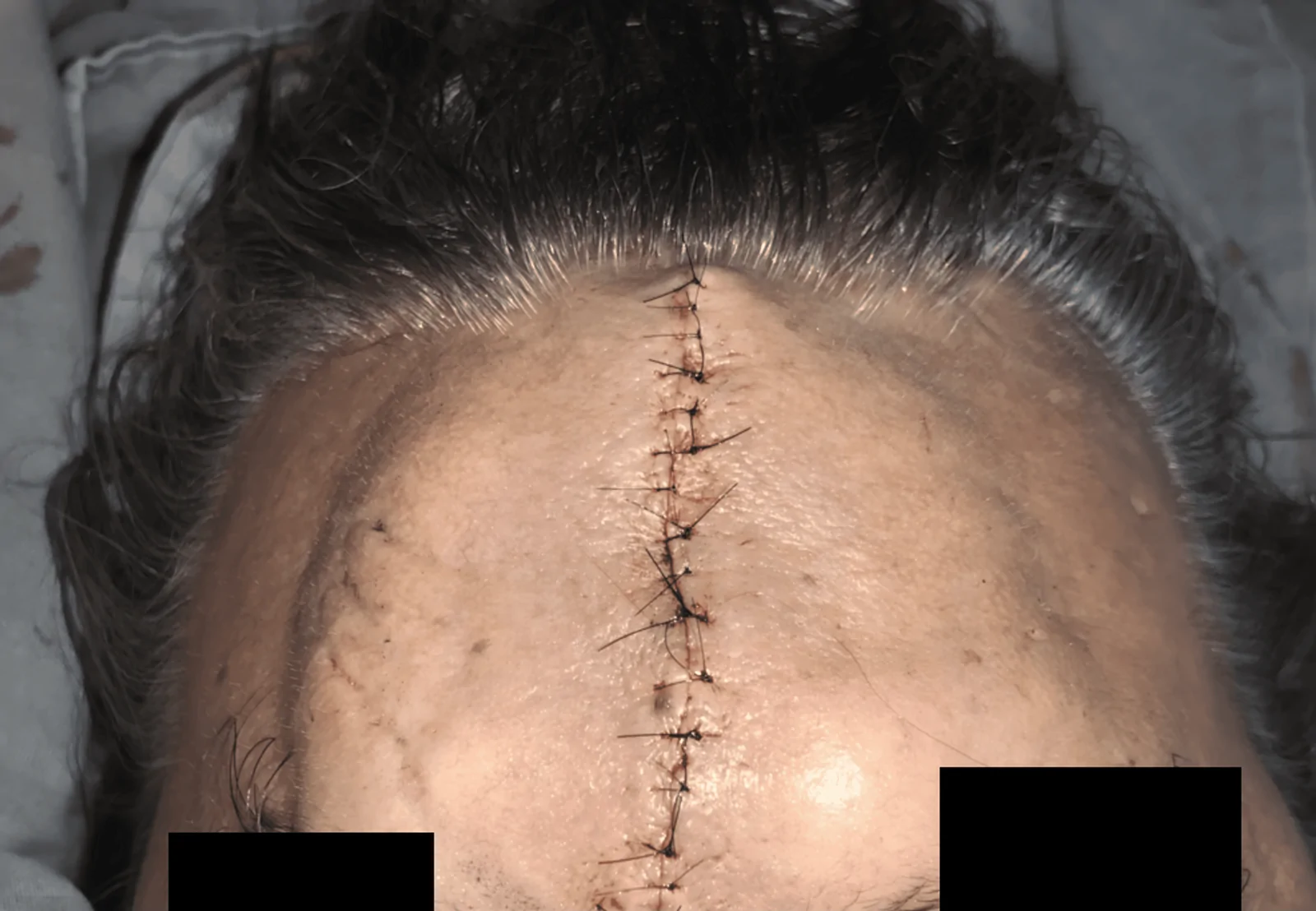
Immediate postoperative appearance following frontal mucocele excision and reconstruction Immediate postoperative frontal view demonstrating closure after drainage of recurrent frontal mucocele, partial capsule resection, excision of redundant frontal skin, and layered wound closure with satisfactory contour restoration.

The procedure consisted of complete drainage of the frontal mucocele followed by partial resection of the capsule to reduce the residual cavity. After evacuation of the mucocele contents, excess capsular tissue was excised, and the remaining capsule was reduced and sutured. Redundant frontal skin and soft tissue resulting from chronic expansion of the lesion were partially excised to improve the forehead contour. The immediate postoperative appearance demonstrated satisfactory decompression of the frontal region and restoration of forehead contour (Figure 3).

**Figure 4:**
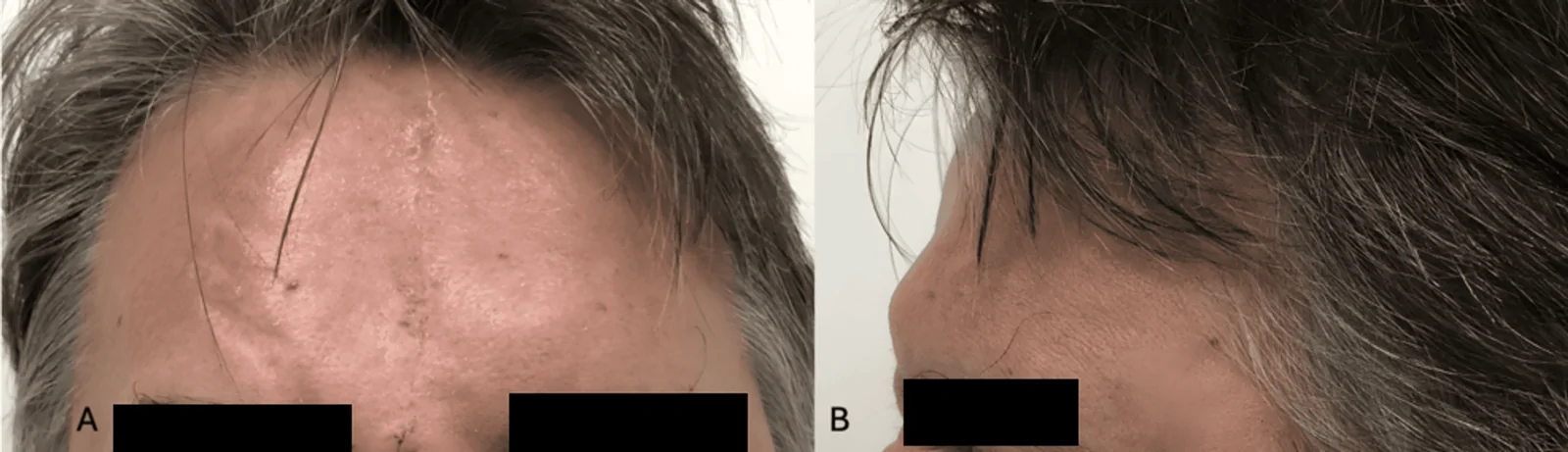
One-year postoperative outcome after surgical treatment of recurrent frontal mucocele (A) Frontal postoperative view at one year demonstrating a well-healed scar, preserved forehead contour, and no clinical evidence of recurrence. (B) Lateral postoperative view at one year showing stable forehead contour without evidence of recurrence.

Histopathological examination of both the drained material and the excised capsule demonstrated no evidence of neoplasia. Serial follow-up showed progressive improvement in forehead contour and scar maturation without recurrence. At one-year follow-up, frontal (Figure 4A) and lateral (Figure 4B) views demonstrated stable restoration of the forehead contour, correction of the frontal projection deformity, favorable scar maturation, and no clinical evidence of recurrent swelling or mucocele recurrence.

## Discussion

Frontal mucoceles are slow-growing lesions that may manifest years after trauma or sinus surgery [1,4]. Obstruction of sinus drainage pathways leads to progressive mucus accumulation and gradual expansion of the sinus cavity. Over time, chronic pressure may result in bone erosion and remodeling, especially in previously traumatized frontal sinuses [3,4].

In the present case, severe prior frontal trauma with loss of the anterior table of the frontal sinus likely contributed significantly to altered sinus drainage and long-term pathological remodeling. In addition, the presence of a frontal osteoma may have further impaired drainage physiology. Osteomas may obstruct the frontal sinus drainage pathway and contribute to secondary mucocele formation, while also increasing the complexity of surgical management [9,12].

Endoscopic treatment is currently regarded as the preferred management strategy for many frontal mucoceles because it avoids external scars and reduces morbidity [2,10,13]. However, recurrence may occur in complex cases involving distorted anatomy, previous surgery, extensive scarring, or post-traumatic alterations [5,13]. In this patient, recurrence developed following previous endoscopic drainage and osteoma shaving, suggesting that the underlying pathological cavity and altered anatomy persisted despite prior intervention.

The surgical strategy used in this case differed from the previously attempted approach. Instead of relying exclusively on drainage and marsupialization, the procedure emphasized reduction of the residual pathological cavity through partial capsule excision and contour correction after complete decompression. The additional management of redundant frontal soft tissue contributed to an improved aesthetic outcome after resolution of the expansile lesion.

The absence of recurrence at one-year follow-up suggests that reduction of residual capsule volume may represent a potential adjunctive strategy in selected recurrent post-traumatic mucoceles, particularly in patients with major anatomical distortion and recurrence following previous endoscopic treatment. However, the independent contribution of this maneuver to recurrence prevention cannot be determined from a single case.

From a reconstructive standpoint, this case also demonstrates the importance of combining functional treatment with aesthetic restoration. Large frontal mucoceles may produce significant facial deformity and psychosocial distress, particularly when recurrence occurs after previous surgery. Addressing redundant soft tissue and contour abnormalities may therefore improve overall patient satisfaction in addition to disease control.

## Conclusions

Recurrent frontal mucoceles following previous endoscopic treatment remain surgically challenging, particularly in patients with severe post-traumatic distortion of frontal sinus anatomy. In this case, complete drainage combined with partial capsule reduction and excision of redundant soft tissue resulted in satisfactory functional and aesthetic outcomes, with stable contour correction and no clinical evidence of recurrence at one-year follow-up. Although these findings are limited to a single patient, this approach may represent a feasible alternative in carefully selected patients with complex recurrent frontal mucoceles and substantial post-traumatic anatomical distortion. Longer follow-up and additional clinical experience are necessary to evaluate the durability and broader applicability of this surgical strategy.
